# Maternally inherited diabetes and deafness with a variable presentation across three generations within a pedigree, South Africa

**DOI:** 10.4102/ajlm.v13i1.2384

**Published:** 2024-05-29

**Authors:** Herbert Makgopa, Tanja Kemp, Surita Meldau, Engela M. Honey, Bettina Chale-Matsau

**Affiliations:** 1 Department of Chemical Pathology, Faculty of Health Sciences, University of Pretoria, Pretoria, South Africa; 2 Department of Chemical Pathology, National Health Laboratory Service, Pretoria, South Africa; 3 Division of Endocrinology, Department of Internal Medicine, Faculty of Health Sciences, University of Pretoria, Pretoria, South Africa; 4 Division of Chemical Pathology, Department of Pathology, University of Cape Town, Cape Town, South Africa; 5 Department of Chemical Pathology, National Health Laboratory Service, Cape Town, South Africa; 6 Division of Human Genetics, Department of Biochemistry, Genetics and Microbiology, University of Pretoria, Pretoria, South Africa

**Keywords:** diabetes, mitochondrial DNA, maternally inherited diabetes and deafness, heteroplasmy, hearing loss

## Abstract

**Introduction:**

Maternally inherited diabetes and deafness (MIDD) is caused by the m.3243A>G pathogenic variant in maternally inherited mitochondrial DNA. Diabetes is prevalent in our setting; however, MIDD is rarely diagnosed. This study, undertaken in Pretoria, South Africa, highlights the variable presentation of MIDD in different patients within the same family.

**Case presentation:**

A 45-year-old man (proband) with hearing impairment was referred to the endocrine unit in July 2015 due to poor glycaemic control (HbA1c = 13%). His clinical and biochemical features were in keeping with MIDD. A genetic study of accessible maternal relatives was pursued. His mother had difficulty hearing and reportedly died from an unspecified cardiovascular cause. Two sisters with diabetes and deafness died of cardiac-related conditions. One nephew had diabetes (HbA1c = 7.7%), hearing loss and tested positive for m.3243A>G. A third sister tested positive for m3243A>G, but aside from bilateral mild hearing loss in higher frequencies, showed no other signs of target organ damage. Her daughter developed end-stage kidney failure necessitating a transplant, while her son had no biochemical abnormalities and was negative for m.3243A>G.

**Management and outcome:**

A multidisciplinary team managed and screened for complications of the patient and his maternal relatives. Proband died prior to genetic testing.

**Conclusion:**

Most MIDD patients initially present with symptoms of diabetes only, and it is probable that many cases remain undiagnosed. A high index of suspicion is necessary when encountering a family history of both diabetes and impaired hearing, and screening should be offered to the patient’s maternal relatives.

**What the study adds:**

This study demonstrates the importance of proper assessment when evaluating a patient with diabetes and a family history of hearing loss.

## Introduction

Primary mitochondrial disease is a heterogeneous group of disorders resulting from inherited defects in mitochondrial energy production.^1^ Maternally inherited diabetes and deafness (MIDD) is a form of primary mitochondrial disease, characterised by diabetes and progressive sensorineural hearing impairment.^2^ It is linked to various pathogenic variants in mitochondrial DNA (mtDNA), including the m.3243A>G point mutation in the *MT-TL1* gene, which has emerged as the most common cause of MIDD, accounting for nearly 85% of all cases.^3^ Other features associated with the m.3243A>G pathogenic variant are myopathy, gastrointestinal tract symptoms, nephropathy, cardiomyopathy, and neuropsychiatric features with heterogeneous manifestation even in individuals within the same family due to varying mtDNA heteroplasmy levels between different tissues, thus developing different phenotypes.

The estimated prevalence of MIDD is between 0.5% and 5.9% among patients with diabetes mellitus.^4^ Diagnosis of MIDD is important to ensure appropriate treatment, continuous monitoring and that maternal relatives are identified and managed in a timeous manner. Diabetes in MIDD, unlike in type 1, initially responds to secretagogues, but may later progress to require insulin for management. Metformin is not suitable in these patients as it may cause or exacerbate lactic acidosis, which is a typical finding in many patients with primary mitochondrial disease.^5^ Maternally inherited diabetes and deafness is rarely diagnosed in clinical practice, and it may be missed due to its complex genetic and phenotypic heterogeneity.^6^

While many studies have been conducted in Europe, Australia, Asia, and North America, MIDD remains severely underdiagnosed on the African continent, with only limited studies available.^7,8,9^ In this context, we describe three generations of South African patients with MIDD within the same pedigree.

## Ethical considerations

Written consent was obtained from the family and ethics approval obtained from the Research and Ethics Committee at the University of Pretoria (No. 496/2023). Confidentiality was ensured by avoiding any use of names, initials, pictures or personal identification numbers in the preparation of this case study.

## Case presentation

The proband, a 45-year-old man, was referred to the endocrine unit of a tertiary hospital in Pretoria, South Africa, in July 2015 due to poor glycaemic control (glycated haemoglobin [HbA1c] = 13%; abnormal > 6.5%). Glycated haemoglobin was assessed using the National Glycohaemoglobin Standardization Program-certified high performance liquid chromatography (Bio-Rad, Hercules, California, United States) method. He was diagnosed with diabetes (presumed type 2), at age 37 years, and hearing impairment was noted a year later. He had a previous admission for congestive cardiac failure and an echocardiogram revealed an ejection fraction of 13% – 15%. At age 42 years, he developed proximal myopathy, leading to reliance on a wheelchair for mobility. He was grossly underweight, with a body mass index of 14.48 kg/m^2^. The patient had established renal impairment (urea = 18.8 mmol/L, reference interval = 2.1–7.1; creatinine = 139 µmol/L, reference interval = 64–104), and an estimated glomerular filtration rate of 47 mL/min per 1.73 m^2^ (chronic kidney disease, stage 3)^10^ with frank proteinuria. Urea was analysed using the urease enzyme assay, creatinine the alkaline picrate while urine protein was tested with the turbidimetric method that utilises benzethonium chloride (Abbott Laboratories, Chicago, Illinois, United States) (Table 1). Type 1 diabetes mellitus was excluded (anti-glutamic acid decarboxylase and islets tyrosine phosphatase 2 antibodies were negative), and C-peptide levels were normal (2.6 µg/L; reference interval = 0.9–7.1), suggesting residual insulin secretion. As C-peptide levels may be decreased in patients with kidney failure, looking for features of monogenic diabetes can assist to reduce misclassification.^11^ He had not experienced any episodes of diabetic ketoacidosis. His clinical and biochemical features were in keeping with MIDD.

**TABLE 1:** Laboratory results of the proband with maternally inherited diabetes and deafness for assessment of complications, obtained between July 2015 and June 2016, South Africa.

Analyte	Patient results	Reference intervals
Thyroid stimulating hormone (mIU/L)	0.67	0.34–5.6
Thyroxine (Free T4) (pmol/L)	13.5	7.6–16.1
Creatine kinase (U/L)	56	20–200
B-type natriuretic peptide (ng/L)	41	0–45
C-peptide (μg/L)	2.6	0.9–7.1
Faecal elastase (μg/g stool)	> 500	> 200
Anti-tissue transglutaminase antibody	Negative	-
Anti-deamidated gliadin antibody	Negative	-
Anti-glutamic acid decarboxylase antibody	Negative	-
Urine protein: creatinine ratio	0.052 g/mmol creatinine	Normal < 0.015 g/mmol creatinine
		Moderate 0.015 g/mmol - 0.05 g/mmol creatinine
		Severe > 0.05 g/mmol creatinine
		Nephrotic syndrome > 0.350 g/mmol creatinine
Urine albumin: creatinine ratio	5.1 mg/mmol creatinine	Normal < 3.0 mg/mmol creatinine
		Moderate 3 mg/mmol - 30 mg/mmol creatinine
		Severe > 30 mg/mmol creatinine

**FIGURE 1:**
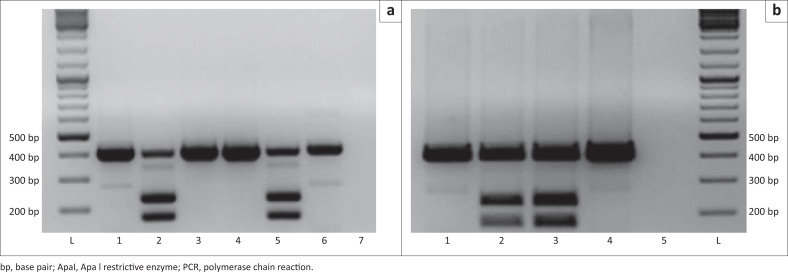
Restriction enzyme digest results from the maternal nephew (III 7) (lane 2, in gel image [a]), and sister (II 4) (lane 2 in [b]) of proband with maternally inherited diabetes and deafness in South Africa, 2016. The m.3243A>G variant creates an ApaI restriction site in the 407 bp PCR amplicon, resulting in 174 bp and 233 bp fragments in affected patients. In unaffected individuals there is 407 bp only (restriction enzyme did not cut) and affected (heteroplasmic) individuals have three bands (wild type [407 bp] and two minor bands [174 bp and 233 bp]). Lanes 1 (both a and b), and 3 and 4 (in a) were other patients that tested negative for the variant. Lane 5 (in a) and lane 3 (in b) are known positive controls. Lane 6 (in a) and lane 4 (in b) are known negative controls. Lane 7 is the no-template control in image a and lane 5 is the no-template control in image b. Lanes L in both images contain GeneRuler DNA Ladder Mix (Thermo Scientific) used as a reference to estimate the size of the unknown fragment.

Genetic investigations were pursued for some of his maternal relatives. Genetic investigation of the proband was not done as the patient demised prior to testing. Only one of his siblings and two of their children were available for testing. The presence of the m.3243A>G variant was investigated using polymerase chain reaction (PCR) followed by the restriction fragment length polymorphism method with ApaI (Thermo Scientific, Waltham, Massachusetts, United States) in DNA extracted from urine samples. Generated fragments were compared against a GeneRuler DNA Ladder Mix (Thermo Scientific, Waltham, Massachusetts, United States) (Figure 1).

**FIGURE 2:**
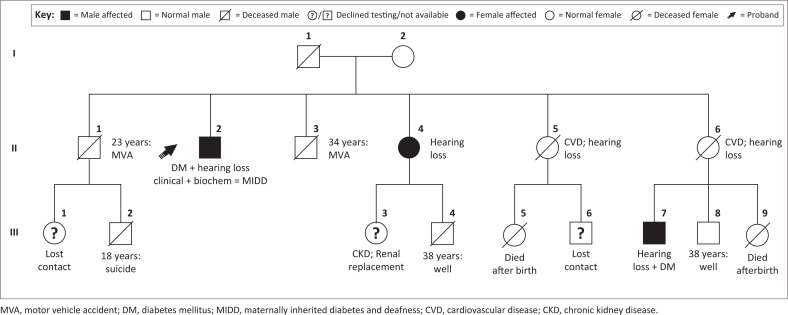
Pedigree of proband’s family with maternally inherited diabetes and deafness showing variable presentation across three generations in South Africa, 2016. While one of the family members (II 4) only developed mild disease later in life, another (III 7) presented with severe disease early in life. The proband’s mother (I 2) and two of his sisters (II 5 and II 6) had hearing loss and all died of cardiovascular related diseases.

The family pedigree (Figure 2) and characteristics (Table 2) indicated variable presentations across three generations. The proband’s mother had hearing difficulties and died from a suspected cardiovascular cause at age 43 years. Among his three sisters, two were diagnosed with diabetes and deafness, and died prematurely, one at age 46 (II 5) due to congestive cardiac failure, and the other at 54 years (II 6) due to acute myocardial infarction. A son of one of these sisters (III 7) was diagnosed with diabetes (HbA1c = 7.7%), hearing loss and had the m.3243A>G variant. The third sister (II 4) also tested positive for the m.3243A>G variant; however, apart from a recent decline in hearing, she had no features of target organ damage. In contrast, her daughter (III 3) developed end-stage kidney failure requiring transplant in her 20s. Her other child, a son (III 4), tested negative for the m.3243A>G variant and has remained free of any related symptoms. Heteroplasmy levels were not determined for any of these cases.

**TABLE 2:** Clinical and laboratory features of proband with maternally inherited diabetes and deafness and available maternal relatives obtained in 2015 and 2016, South Africa.

Clinical and laboratory features	Normal values	I 2 (Mother)	II 2 (Proband)	II 4 Sister	III 3 Niece	III 4 Nephew	III 7 Nephew
**Clinical characteristics**							
Age (years)	-	43 [at death]	45	57	25	35	35
Gender	-	Female	Male	Female	Female	Male	Male
Hearing impairment	-	Yes	Yes	Yes	No	No	Yes
Diabetes diagnosis	-	Unknown	Yes	No	ND‡	No	Yes
Alive/Deceased	-	Deceased	Deceased	Alive	Alive	Alive	Alive
**Laboratory results**	-						
HbA1c%	< 6.5%	ND†	13	ND‡	ND‡	5.5	7.7
Urea	2.1 mmol/L – 7.1 mmol/L	ND†	20	6.0	ND‡	6.0	6.1
Creatinine	Male: 64 μmol/L – 104 μmol/L; Female: 49 µmol/L – 90 µmol/L	ND†	166	61	ND‡	83	100
MDRD	> 60 mL/min per 1.73 m^2^	ND†	39	> 60	ND‡	> 60	> 60
Creatine kinase	20 U/L – 200 U/L	ND†	56	ND‡	ND‡	ND‡	103
*MT-TL1* m.A3243G variant	-	ND†	ND†	Detected	ND‡	Absent	Detected

ND, not done; HbA1c, glycated haemoglobin; MDRD, modification of diet in renal disease; *MT-TL1*, mitochondrially encoded transfer ribonucleic acid leucine 1.

† , laboratory test was not performed, because no sample was available (individual was deceased);

‡ , laboratory test was not performed, because individuals were not available for sample collection.

## Management and outcome

A multidisciplinary team was involved in the management of the patients discussed in this study. Insulin secretagogues were considered for the treatment of diabetes. The proband had severe myopathy requiring a wheelchair. The patient was mostly managed in primary care, and was referred very late to a specialist when he had already developed severe complications, including cardiovascular issues. He sustained a femur fracture with presumptive pulmonary embolism and died before genetic confirmation could be obtained. His maternal relatives were offered genetic counselling and screening for diagnosis and management of complications.

## Discussion

The pathogenic variant m.3243A>G of the *MT-TL1* gene is one of the identified causes of mtDNA-related primary mitochondrial disease worldwide. It was initially identified in patients with mitochondrial encephalopathy, lactic acidosis and stroke-like episodes in 1990 and a couple of years later in a large pedigree with maternally transmitted diabetes mellitus and deafness.^12^

The exact mechanism by which the m.3243A>G variant causes disease is still incompletely understood. However, decreases of pancreatic beta cell mass and declines in glucose-stimulated insulin secretion have been implicated in the development of diabetes in affected patients.^13^ Maternally inherited diabetes and deafness is exclusively inherited through the maternal line, as the paternal contribution of sperm mitochondria to the fertilised egg is minimal, about 1000-fold less than that of the oocyte, and there are selective mechanisms targeting paternal mtDNA for destruction, further reinforcing the maternal inheritance pattern.^14^

Patients with MIDD range in age from 12 years to 67 years when they present with diabetes, and diabetes is often the presenting problem, although in some patients, onset of sensorineural deafness may precede presentation of diabetes.^13^ A definitive diagnosis is only possible through genetic confirmation. Screening for the common m.3243A>G variant is best performed using DNA from epithelial cells in urine or muscle, as heteroplasmy levels tend to decrease with age in actively dividing cells in blood.^15^ In the case of the proband, the MIDD diagnosis was inferred from a combination of clinical and laboratory features, a strong family history, and the positive identification of the m.3243A>G variant in some of his maternal relatives.

The clinical spectrum of MIDD exhibits heterogeneity, encompassing variation in terms of age of onset, clinical manifestation and severity.^5^ As observed in this study, the proband’s sister tested positive for the m.3243A>G pathogenic variant but remained asymptomatic throughout most of her adult life. She only developed decreased hearing after reaching age 60 years.

Complications in patients with MIDD follow a distinct pathophysiology compared to complications in patients with type 1 and type 2 diabetes. Proteinuria arises as a consequence of the mitochondrial dysfunction.^2,5^ The renal impairment is frequently misdiagnosed as diabetic kidney disease; however, in some patients, kidney failure may even develop before the onset of diabetes. As shown with the proband’s niece, end-stage kidney disease necessitating kidney transplantation can develop at a young age.

Pathogenic mtDNA variants, such as the m.3243A>G variant, tend to be heteroplasmic in all affected individuals. One of the proband’s nephews tested negative for this variant, but had no features of end-organ damage. These diverse patterns of heteroplasmy underscore the complex nature of mitochondrial genetics and their impact on phenotypic expression.

In this study, the PCR-restriction fragment length polymorphism method employed is still used in clinical laboratories for detection of the m.3243A>G variant. However, it is unable to detect heteroplasmy levels below approximately 5% and is not quantitative.^15^ In contrast, methods such as next generation sequencing, pyrosequencing, real-time PCR and ligation-mediated PCR have the advantage of accurately detecting much lower heteroplasmy levels,^16^ although due to their higher costs, these techniques are not routinely available in most public clinical laboratories.

There is no cure for MIDD, and treatment mainly focuses on managing disease progression and minimising severity of complications in order to enhance the quality of life for affected individuals.^13^ As is evident from this study, cardiac abnormalities are common in patients with MIDD and can contribute to premature death. Thus, echocardiography and regular electrocardiography should form part of the standard evaluation for MIDD.

Optimal patient management in MIDD requires a multidisciplinary approach, including supportive therapy and genetic counselling. These should be offered to all available maternal relatives to provide information on the mode of inheritance and enable informed choices and considerations for family planning.^17^

Considering that symptoms of diabetes are often the reason for the initial health-seeking behaviour in most MIDD patients, it is probable that many cases remain undiagnosed in our local setting. A high index of suspicion should be maintained when encountering a family history of diabetes and impaired hearing. All maternal offspring and relatives of an individual with MIDD are potential carriers of a pathological variant and should be offered screening.
